# Evaluation of Cadmium or Lead Exposure with *Nannochloropsis oculata* Mitigation on Productive Performance, Biochemical, and Oxidative Stress Biomarkers in Barki Rams

**DOI:** 10.1007/s12011-022-03318-z

**Published:** 2022-06-16

**Authors:** Marwa A. Hassan, Yasmina K. Mahmoud, A. A. S. Elnabtiti, A. S. El-Hawy, Moharram Fouad El-Bassiony, Heba M. A. Abdelrazek

**Affiliations:** 1 Department of Animal Hygiene, Zoonoses and Behavior, Faculty of Veterinary Medicine, Suez Canal University, Ismailia, 41522 Egypt; 2 Biochemistry Department, Faculty of Veterinary Medicine, Suez Canal University, Ismailia, 41522 Egypt; 3 Animal Wealth Development Department, Faculty of Veterinary Medicine, Suez Canal University, Ismailia, 41522 Egypt; 4 Animal and Poultry Production Division, Desert Research Center, Cairo, Egypt; 5 Department of Physiology, Faculty of Veterinary Medicine, Suez Canal University, Ismailia, 41522 Egypt

**Keywords:** *Nannocloropsis oculata*, Cadmium, Lead, Triiodothyronine, Thyroxin, Liver enzymes, Cholesterol

## Abstract

This study was designed to determine the lead or cadmium exposure of Barki rams and the beneficial role of *Nannochlorposis oculata* (*N. oculata*) 4% as a feed supplement, as well as its mitigating role against these elements’ impacts concerning performance, biochemical markers of liver enzymes and kidney function, thyroid hormone activity, and oxidative stress markers. Six groups of 36 Barki rams (33.63 ± 1.29 kg) were divided into G1: which served as control; G2: was given 4% dietary *N. oculata*; G3: was given oral 1 mg/kg cadmium chloride; G4: was given 5 mg/kg/day lead acetate; G5: was given oral 1 mg/kg cadmium chloride and 4% dietary *N. oculata*, and G6: was given oral 5 mg/kg/day lead acetate and 4% dietary *N. oculata*; and treatments were continued for 60 days. Cadmium and lead-exposed groups exhibited lower and weaker weight gain as well as feed conversion ratio, respectively, than the control and other groups. Additionally, levels of T3, T4, total proteins, albumin, and glutathione (GSH) were significantly reduced in both G3 and G4 compared to control. However, urea, creatinine, ALT, AST, total cholesterol, triglycerides, protein carbonyl content (PCC), and malondialdehyde (MDA) were significantly increased (*P* ≤ 0.05) in cadmium and lead-exposed groups. Dietary *N. oculata* (4%) improves serum proteins, creatinine, urea, T4, and oxidative stress indicators as compared to the control group. Finally, 4% dietary *N. oculata* greatly enhances the investigated parameters in terms of performance, thyroid hormones, serum biochemical, and antioxidant activity and may assist in reducing the endocrine disrupting effects of Pb and Cd*.*

## Introduction

Heavy metal toxicity is growing in developing countries as urbanization and industrialization proceed [1]. The most major environmental and industrial contaminants have been identified as lead (Pb) and cadmium (Cd). Their pollution coexists with humans and animals in a variety of situations [2]. Cadmium is among the most toxic minerals in the ecosystem to animals and humans [3, 4] with organ toxicity ranging from mild to severe and a long half-life of clearance [5], which is not essential to physiological and biochemical functions [4]. It occurs naturally with greater concentrations in Cd-rich soils such as shales, marine and lacustrine sediments, and phosphorites. Nevertheless, industrial and agricultural processes account for more than 90% of Cd in the surface environment [6]. Cd exposure in farmed ruminants occurs due to industrial processing and intensive agricultural practices that pollute water, soil, forage, feed, and air [7]; also, it is a phosphate fertilizer pollutant [8]; therefore, it is introduced to the soil via routine farming practices [9]. The highest Cd concentration tolerated in animal diets is 0.5 mg kg.^−1^ [10]. Whenever animals consume substantial quantities of Cd, it can bioaccumulate for decades, resulting in subacute, acute, or chronic intoxication [4], causing significant damage to numerous organs such as the liver and kidney, as well as structural and physiological abnormalities [4, 10]

Lead has a negative influence on animal health and production due to its inability to degrade and bio-accumulate over long periods of time [11], affecting all biological systems through exposure from water, food sources, and air [12]. Pb is recognized as a crucial ecological pollutant that has been linked to unintentional toxicity in domestic animals, most commonly in industrialized areas of the world [13, 14]. Pb intoxication in animals is also commonly caused by contaminated feed from industrial effluents, home wastes, fertilizers, pesticides, and mineral combinations [15, 16]. Furthermore, the feed may be polluted by vehicle oil, pastures close to Pb industrial facilities and battery factories, ash from oil-painted wood, lubricants from machines, disposed paint cans, plies [17, 18], and railings, walls, floors, drinkers, feeders, and storage facilities with Pb-containing paints that animals might indeed lick [17, 19]. Another source is meadows along road boundaries that have been polluted with high quantities of fumes emitted by gasoline automobiles since their fuel includes Pb tetraetileno [20]. Water pollution by hazardous heavy metals such as Pb has increased rapidly as a result of natural and industrial sources [21], and these metals have subsequently reached plants and animals, which have a significant impact on human health via the food chain [22]. Pb exposure even in a small dose for a long period causes clinicopathological alterations due to damage to the liver, kidney, endocrine system, and reproductive performance of animals [23, 24].

The gap between growing water demand and restricted water availability is Egypt’s most serious water resource management concern. The new land projects need large volumes of water, which can only be obtained by improving water irrigation efficiency on previously watered old lands, as well as reusing drainage water and purified wastewater [25]. El-Salam Canal is one of the five enormous irrigation projects under construction in Egypt’s Northern Sinai. It has an impact on animal health and productivity. The Egyptian government plans to restore an estimated 620,000 ac of desert along Sinai’s Mediterranean coast by redirecting considerable volumes of agricultural drainage water to newly reclaimed regions and mixing it in a 1:1 ratio with Nile water [26]. El-Salam canal (latitudes 32° 40′ to 44°, longitudes 31° 40′ to 16°) runs southeast towards Lake El-Manzala, then south to mingle with El-Serw drainage water in a 1:1 ratio, then east, then south to combine with Hadous drainage water, then east beneath Suez Canal to Sinai peninsula [27]. Nevertheless, water contamination, which is a major environmental problem, may emerge from this mixing of water [26]. Furthermore, it is polluted by a wide range of pollutants, including elevated levels of minerals, heavy metals, organic debris, pesticide and herbicide residues, and microbiological contamination [28]. Multiple investigations identified cadmium and lead contamination in the El-Salm canal at higher levels of 0.215 to 2.17 and 5.20 mg/l, respectively [25], and metal concentrations in water fluctuated between years (2015–2018) and were Cd (0.76–0.87), Pb (0.98–1.12) mg/l, respectively [29]. The most widespread ruminant livestock species, grazing sheep, is one of Egypt’s agricultural foundations because it can convert low-quality roughages into meat and milk for human use, in addition to generating wool and hide [30]. The most widespread ruminant livestock species, grazing sheep, is one of Egypt’s agricultural foundations because it can convert low-quality roughages into meat and milk for human use, in addition to generating wool and hide [31]. The suitability and availability of key macro and microelements from pastures influence grazing animal performance and health. Animals under this regime rely entirely on forages to meet all their nutritional requirements. Metals and metalloids, for example, are hazardous chemicals or compounds that accumulate throughout the food chain. Furthermore, their concentrations in the environment rise in response to increases in urban, agricultural, and industrial emissions. The extensive prevalence of some metal pollutants, notably Cd and Pb, allows them to enter the food chain, raising the likelihood of harmful effects on people and livestock [32]. Additionally, the latter authors found Cd and Pb (0.54–0.8 and 3.32 to 5.76 mg/l, respectively) in fodder grown in the East Qantara area, near the El-Salam canal.

Natural antioxidants used as nutritional supplements, including microalgae, may enhance not only the health and performance of animals but also their resilience to environmental stressors such as heat stress, poor housing conditions, and infections [33]. Microalgae have previously been reported as an alternative non-traditional protein source and nutritional supplement for animal and human nutrition, but commercial large-scale production began just a few decades ago [34]. *Nannochloropsis* species have grown in popularity as a source of lipids for biofuels and/or the synthesis of long-chain polyunsaturated fatty acids, notably eicosapentaenoicaci [35]. Commercialization of this alga is being pursued [36]. *Nannochloropsis* species are freshwater and marine microalgae that are linked to diatoms and brown alga*e* [37], they have been utilized to make nutraceuticals and feed additives for decades [38]. *Nannochloropsis* sp. has been used as an aquaculture feed ingredient, providing a supply of omega-3 fatty acids [39]. Among the microalgae that should be included as a feed supplement, *Nannochloropsis* species should be prioritized due to their suitability for intensive cultivation and high concentration of PUFAs (particularly EPA), antioxidants, and certain vitamins [34]. Additionally, *Nannochloropsis oculata* (*N. oculata*) is a marine-water microalga that is a strong source of omega-3 fatty acids, notably eicosapentaenoic acid (EPA), which is used to make an omega-3 oil for use as a dietary supplement [40]. According to Altomonte et al. [41], ruminants are good models for feeding with microalgae since they can break down cell wall organisms that are typically not metabolized. Kholif et al. [42] concluded that microalgae in diets improved feed utilization, milk production and quality, productive performance, and meat quality in ruminants because of improved diet nutritive value, leading to improved feed utilization; conclusively, feeding Nubian goats on a diet containing *N. oculata* (5 and 10 g) improved milk production and the nutritive value of the diet.

In this respect, this study aimed to investigate the lead or cadmium exposure on Barki rams, as well as the beneficial role of *N. oculata* 4% as a feed supplement and its moderating role against these elements’ impact. This was accomplished by monitoring their performance, biochemical indicators of liver enzymes and renal function, thyroid hormone activity, and oxidative stress markers.

## Materials and Methods

All the experimental methods were carried out by well-trained experts in conformity with the principles of Suez Canal University’s Animal Ethics Review Committee.

### Animals and Experimental Design

The experiment was carried out on 36 healthy Barki rams (*n* = 6/group), aged approximately 6 months and weighing a mean of 33.63 ± 1.29 kg, which were raised for 60 days. The animals were housed in freely ventilated semi-closed pens with partitions between groups throughout the experimental period, with water supplied by troughs and shade provided for sun protection at the sheepfold of a private farm near the El-Salam canal in Sahl Altina, East Qantra area, Ismailia, Egypt. Before commencing the experiment, feces samples were submitted for parasitological analysis to determine the health of the animals [43]. Clinical examination of the animals (body temperature, mucous membrane, respiratory rate, pulse rate, and ruminal motility of all animals) was monitored according to Kelly [44]. The control sheep were offered feed and water free from Cd and Pb. The animals were divided into 6 groups: Control (G1): animals received basal diet only; Nanno. group (G2): animals received basal diet with 4% dietary *N. oculata*; Cd (G3): animals administrated Cd chloride (1 mg/kg/day); Pb (G4): animals administrated Pb acetate (5 mg/kg/day); Nanno + Cd (G5): animals administrated Cd chloride (1 mg/kg/day) and 4% dietary *N. oculata*; and Nanno + Pd; animals administrated Pb acetate (5 mg/kg/day) and 4% dietary *N. oculata*. Based on the levels detected in forage by Donia and Marwa [32], heavy metals were administrated to the experimental sheep, Cd (1 mg/kg/ day) [45] and Pb acetate (5 mg/kg/day) [46] orally for 60 days. The concentration of heavy metals was calculated by the following equation (Eq. (1)):

$$ppm\;of\;element=\frac{Molecular\;weight\;of\;salts}{Molecular\;mass\;of\;the\;element}\times required\;Conc.of\;elements$$

Cadmium and lead (99.99% purity CdCl2 and lead (II) acetate trihydrate, Merck) were administered to the animals. By dissolving cadmium chloride (0.163 g) and lead acetate (0.915 g) (Eq. (1)) in 1 l of distilled water, a stock solution of 500 and 100 mg l^−1^ of Cd and Pb was prepared, with the final concentration reaching 100 × (100 and 500 mg/l for cadmium and lead, respectively). The desired concentration (1 and 5 mg l^−1^ for Cd and Pb, respectively) was then obtained by adding 0.1 ml of the stock solution to 9.9 ml of distilled water, then the 10 ml was administered to the animals orally onto the tongue using disposable plastic syringe with a rubber long nozzle and the fluid was readily swallowed by the rams. The control group received the same treatment as the other groups, but with distilled water instead of heavy metals, and was kept under the same conditions.

### Experimental Diet and Microalga

The basal diet was designed to suit the ram’s nutritional requirements while balancing body weight gain at a rate of 0.3 kg/day [47]. The composition of the basal diet is presented in Table 1. Diet was provided twice a day, in the morning and evening, with unrestricted access to water. On days 0 and 60 of the experiment, animals were weighed after fasting for 12 h prior to the morning feedings. The Algal Biotechnology Unit, Biological and Agricultural Research Division, National Research Centre, Dokki, Giza, Egypt, cultivated and retrieved *Nannochloropsis oculata* (NNO-1 UTEX Culture LB 2164). The algae meal was added to the concentrate in the mixer at the feed mill. The concentrate intake was calculated from feed offered and refused on daily basis. Before the experiment, Cd and Pb levels in the diet, water, and *N. oculata* were measured and found to be undetectable.

**Table 1:** Ingredients and calculated chemical composition of experimental diet

Item	%	Analyzed chemical composition (% on DM basis)	
Ingredient			
Berseem Hay	14.65	DM	88.7
Wheat straw	4.88	CP	14.2
Corn grains	57.7	TDN	76.2
Cotton seed meal	13.07	NDF	22.8
Soybean meal	7.4	ADF	14.9
Limestone	0.81	EE	4.14
Salt	0.50	Ca	0.7
Sodium bicarbonate	0.51	P	0.4
Ammonium chloride	0.20		
Vitamin-mineral premix	0.30		

*DM* dry matter; *CP* crude protein; *TDN* total digestible nutrient; *NDF* neutral detergent fiber; *ADF* acid detergent fiber, *EE* ether extract; *Ca* calcium; *P* phosphorus

### Growth Performance Parameters

Each group’s rams were weighted at the start (initial body weight) and end (final body weight) of the trial. Furthermore, overall weight growth (kg/head) and average daily gain (g/head/day) were estimated. Feed intake (g DM/head) was recorded daily, and the feed conversion ratio (FCR kg DM/kg gain) was determined to evaluate the performance of the rams [48].

### Blood Sample Collection and Biochemical Parameters

At 60 days of the trial, blood samples of 10 ml were collected from the jugular vein into sterile vacutainer tubes guaranteed free of any trace of heavy metals to harvest serum for biochemical studies. Between the hours of 8 and 9 a.m., blood samples were obtained. After 30 min at ambient temperature, blood samples were centrifuged at 3000 rpm for 15 min, and the sera were stored at − 20 °C until analysis.

Serum triiodothyronine (T3) and thyroxin (T4) concentrations were determined by radioimmunoassay (RIA) [49]. Serum proteins (total, albumin, globulin, and A/G ratio) and serum globulin concentrations were calculated by the difference between total protein and albumin concentrations. alanine aminotransferase (ALT), aspartate aminotransferase (AST), and serum creatinine, urea levels, cholesterol, and triglycerides were measured by using UV/visible spectrophotometer; test procedures were performed as per the manufacturer’s instructions (Diamond Diagnostic, Egypt), according to the method described by Young and Friedman [50].

Cd and Pb levels in serum samples were determined using atomic absorption spectrophotometry (Thermo Electron Corporation, model S4AA sys. USA) at a wavelength of 228.8 nm and 283.3 nm, respectively [51].

### Oxidative Stress Markers

The changes in MDA levels in the serum samples as an endpoint of lipid peroxidation were calculated by detecting the absorbance of thiobarbituric acid reactive substances at 532 nm [52]. Glutathione (GSH) levels were determined by measuring absorbance at 412 nm [53]. Estimation of protein carbonyl content (PCC) was based on the reaction between 2,4-dinitrophenylhydrazine (DNPH) which was analyzed spectrophotometrically at an absorbance of 370 nm. The above-mentioned parameters were measured using a commercially available kit following the manufacturer’s instructions.

### Statistical analysis

The data were processed using the SPSS version 22 computer program (Inc., 1989–2013), and the results are displayed as means ± SE for each treatment, with a one-way ANOVA analysis of variance LSD test conducted to test for a significant difference between treatments at *p* ≤ 0.01. The principal component analysis (PCA) method published by Liu et al. [54] was used for factor analysis.

## Results

### Growth Performance Parameters

All the animals tested in the different treatments, as well as the control group, demonstrated normal clinical parameters (data not shown). According to the statistics, the starting body weight was nearly identical. The experimental groups’ values of dry roughage intake, concentrate intake, and total DMI (g/kg BW) were all negligible (Table 2). Animals exposed to Cd or Pb had poor growth performance, as evidenced by a significant (*P* ≤ 0.05) decrease in weight gain and a significant (*P* ≤ 0.05) weak FCR when compared to other groups (Table 2). Adding *N. oculata* 4% to the experimental diet of Pb and Cd intoxicated rams significantly (*P* ≤ 0.05) improved weight gain and FCR than Cd and Pb groups; however, final body weights of the latter groups were not non-significantly varied. The addition of *N. oculata* 4% to diet in the control negative group (G2) exerted no influence on the final body weight and weight gain.

**Table 2:** Growth performance and feed conversion ratio among the treated groups

Parameters	Body weight changes				Daily feed intake (g DM/head)			
Treatments (*n* = 6/group)	Initial BW (kg)	Final BW (kg)	Total gain (kg/head)	Average daily gain (g/head/day)	Concentrate mixture	Roughages	Total DM intake/head/day	FCR (kg DM/kg gain)
Control	34.5^a^ ± 1.8	43.87^a^ ± 1.67	9.37^ab^ ± 0.28	156.11^ab^ ± 4.73	877.33^a^ ± 33.47	658^a^ ± 25.1	1535.33^a^ ± 58.57	9.9^d^ ± 0.55
Nanno	32.5^a^ ± 3.31	42.64^a^ ± 3.13	10.14^a^ ± 0.5	169.06^a^ ± 8.37	852.87^a^ ± 62.62	639.65^a^ ± 46.96	1492.52^a^ ± 109.58	8.99^d^ ± 0.88
Cd	34.67^a^ ± 4.58	39.55^a^ ± 3.94	4.89^d^ ± 0.66	81.44^d^ ± 11.05	791.07^a^ ± 78.78	593.3^a^ ± 59.08	1384.37 ^a^ ± 137.86	19.69^ab^ ± 4.15
Pb	32.33^a^ ± 3.41	37.34^a^ ± 3.03	5.01^d^ ± 0.49	83.5^d^ ± 8.14	746.87^a^ ± 60.54	560.15^a^ ± 45.41	1307.02^a^ ± 105.95	16.92^bc^ ± 2.81
Nanno + Cd	34.33^a^ ± 4.05	41.88^a^ ± 3.94	7.54^c^ ± 0.97	125.72^c^ ± 16.2	837.53^a^ ± 78.88	628.15^a^ ± 59.16	1465.68^a^ ± 138.04	12.93^ cd^ ± 2.14
Nanno + Pb	33.42^a^ ± 2.29	41.1^a^ ± 2.21	7.68^bc^ ± 0.52	128.06^bc^ ± 8.7	822^a^ ± 44.13	616.5^a^ ± 33.1	1438.5^a^ ± 77.23	11.47^ cd^ ± 0.9

Differences between means within the same column having different superscript letters are statistically significant (*P* ≤ 0.05)

*BW* body weight, *DM* dry matter, *FCR* feed conversion ratio

### Thyroid Hormones and Biochemical Parameters

**Fig. 1:**
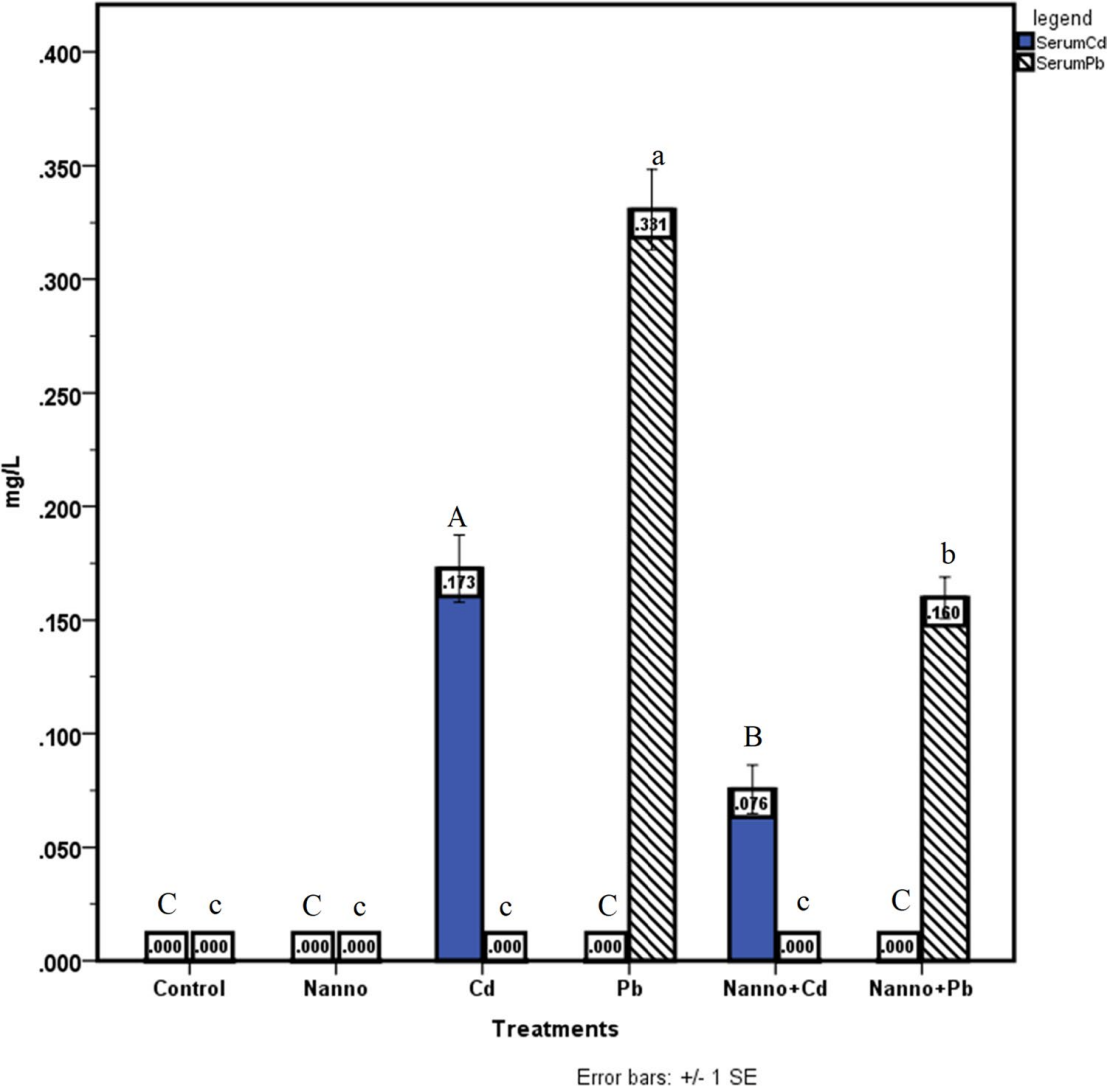
Serum levels of Cd and Pb among the exposed groups. Differences between means having different superscripts (capital letters for Cd and small letters for Pb) are statistically significant (*P* ≤ 0.001)

Before exposure, mean blood Cd and Pb levels were below the detection limit (5 µg l^−1^) in all groups. Serum Cd levels in G3 and G5 (0.173 ± 0.02 and 0.076 ± 0.01 mg l^−1^, respectively) were significantly different (*P* ≤ 0.001). While serum Pb was identified at the following levels in G4 and G6, respectively, with a statistical variation (*P* ≤ 0.001) of 0.331 ± 0.02 and 0.160 ± 0.01 mg l^−1^. Cd and Pb were not detected in either G1 or G2 (Fig. 1).

Thyroid hormones (T3 and T4) results are shown in Table 3. The Cd and Pb exposure resulted in a significant (*P* ≤ 0.0001 and 0.01, respectively) reduction of T3 and T4 serum levels than control. Administration of dietary *N. oculata* 4% to Cd- and Pb-exposed rams caused significant (*P* ≤ 0.0001) improvement of T3 and T4 levels when compared to Pb- and Cd-exposed rams. In regard to biochemical parameters, data shown in Table 3 demonstrate significant (*P* ≤ 0.05) increases in creatinine (mg dl^−1^), urea (mg dl^−1^), ALT (U L^−1^), AST (U L^−1^), cholesterol (mg dl^−1^), and triglycerides (TG) (mg/dl) in Cd- and Pb-intoxicated rams, as compared to control. On the other hand, significant (*P* ≤ 0.001) hypoproteinemia associated with hypoalbuminemia was observed in Cd- and Pb-administered groups, as compared to the control rams. *N. oculata* 4% as a Cd or Pb toxicity mitigator. Treated animals with *N. oculata* 4% in combination with Cd (G5) or Pb (G6) demonstrated a significant (*P* ≤ 0.05) improvement in all the later evaluated parameters when compared to rams exposed to heavy metal toxicity (G3 and G4). Concerning the effect of *N. oculata* 4%, as compared to the negative control group, non-significant changes were recorded in all examined parameters except T4, total protein, albumin, and globulin that were significantly increased than the control. Moreover, rams in G2 (Nanno 4%) had the highest significant (*P* ≤ 0.01) serum total protein, albumin, and globulin levels among the experimental groups. The levels of urea and creatinine were significantly (*P* ≤ 0.05) reduced in the *N. oculata* 4% group (G2) than the control (G1).

**Table 3:** Thyroid hormones and biochemical parameters among the treated groups

Treatments (*n* = 6/group)	Control	Nanno	Cd	Pb	Nanno + Cd	Nanno + Pb	Sig. *P* ≤
T3 (ng/ml)	0.91^**a**^ ± 0.03	0.91^**a**^ ± 0.02	0.41^**c**^ ± 0.01	0.46^**c**^ ± 0.03	0.71^**b**^ ± 0.04	0.74^**b**^ ± 0.03	0.0001
T4 (ng/ml)	9.08^**b**^ ± 0.3	10.8^**a**^ ± 0.45	3.99^**d**^ ± 0.14	4.98^**d**^ ± 0.3	7.03^**c**^ ± 0.37	7.09^**c**^ ± 0.34	0.01
Creatinine (mg/dl)	1.63^**c**^ ± 0.07	1.32^**d**^ ± 0.03	2.33^**a**^ ± 0.15	2.07^**ab**^ ± 0.21	1.82^**bc**^ ± 0.05	1.59^**c**^ ± 0.06	0.05
Urea (mg/dl)	21.04^**d**^ ± 0.99	17.74^**e**^ ± 0.82	49.22^**a**^ ± 1.05	44.66^**b**^ ± 1.28	32.4^**c**^ ± 1.74	29.4^**c**^ ± 1.56	0.05
ALT (U/l)	19.15^**c**^ ± 0.55	18.28^**c**^ ± 0.48	34.48^**a**^ ± 0.91	32.78^**a**^ ± 0.94	27.08^**b**^ ± 0.58	25.13^**b**^ ± 0.69	0.0001
AST (U/l)	31.28^**c**^ ± 0.34	29.8^**c**^ ± 0.5	47.57^**a**^ ± 0.99	45.05^**a**^ ± 1.29	36.52^**b**^ ± 0.98	34.74^**b**^ ± 1.22	0.01
Cholesterol (mg/dl)	45.58^**c**^ ± 1.27	39.11^**c**^ ± 0.96	100.09^**a**^ ± 5.95	96.67^**a**^ ± 5.46	66.68^**b**^ ± 2.79	70.2^**b**^ ± 2.76	0.0001
TG (mg/dl)	32.23^**c**^ ± 1.24	26.89^**c**^ ± 0.48	71.73^**a**^ ± 2.94	68.21^**a**^ ± 4.48	49.13^**b**^ ± 2.34	48.52^**b**^ ± 2.2	0.0001
Total protein (g/dl)	6.8^**b**^ ± 0.11	7.9^**a**^ ± 0.06	4.89^**d**^ ± 0.13	4.94^**d**^ ± 0.16	6.19^**c**^ ± 0.16	6.17^**c**^ ± 0.16	0.001
Albumin (g/dl)	4.66^**b**^ ± 0.08	5.29^**a**^ ± 0.07	2.71^**c**^ ± 0.2	2.87^**c**^ ± 0.19	4.31^**b**^ ± 0.09	4.28^**b**^ ± 0.1	0.0001
Globulin (g/dl)	2.14^**b**^ ± 0.06	2.6^**a**^ ± 0.07	2.18^**b**^ ± 0.17	2.07^**b**^ ± 0.17	1.88^**b**^ ± 0.14	1.89^**b**^ ± 0.15	0.01
A/G ratio	2.2^**a**^ ± 0.077	2.06^**a**^ ± 0.083	1.31^**b**^ ± 0.194	1.44^**b**^ ± 0.161	2.37^**a**^ ± 0.201	2.34^**a**^ ± 0.2	0.01

Differences between means within the same row having different superscript letters are statistically significant at ***P* ≤ **0.0001, 0.01, 0.05

### Oxidative Stress

**Fig. 2:**
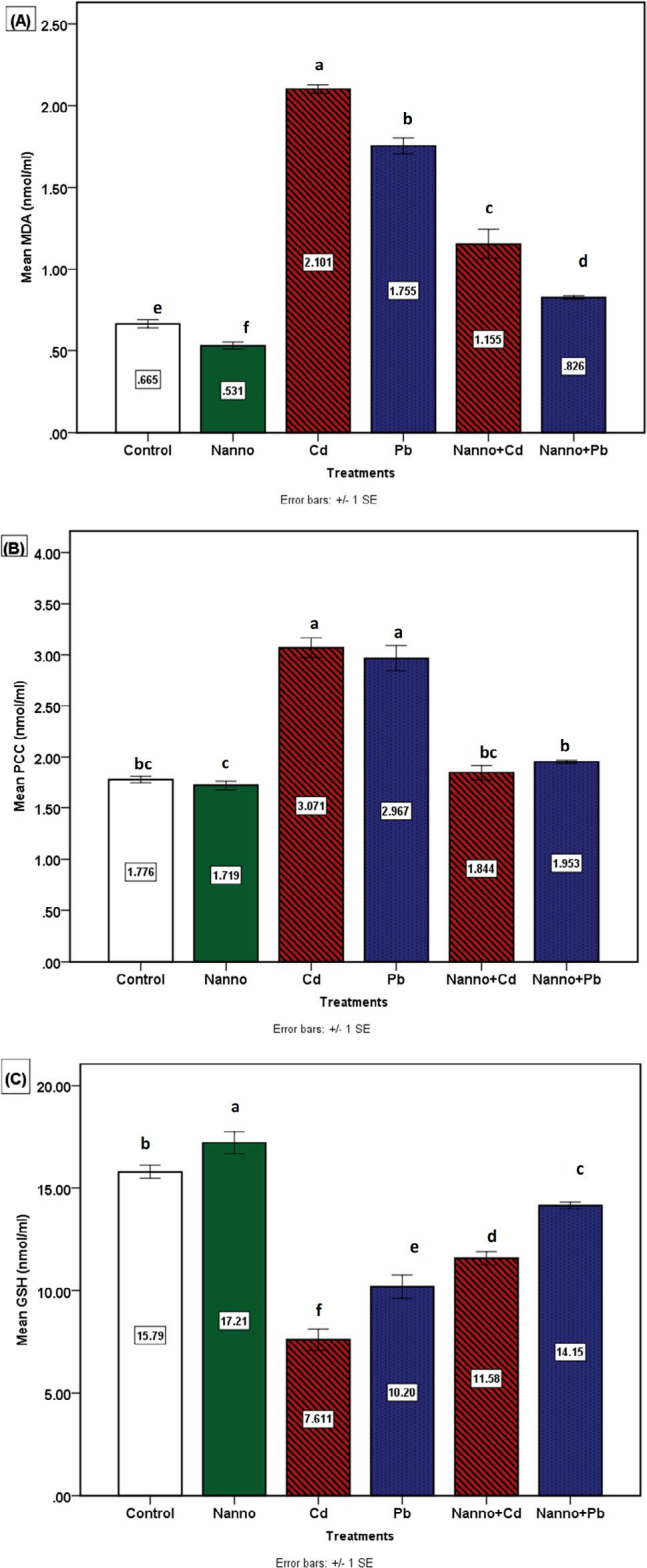
Oxidative stress markers among the treated groups. (A) Malondialdehyde (MDA) nmol/ ml. (B) Protein carbonyl content (PCC) nmol/ml. (C) Glutathione (GSH) nmol/ml. Differences between means having different superscript letters are statistically significant (*P* ≤ 0.05)

The administration of Cd (G3) and Pb (G4) to experimental rams resulted in a significant (*P* ≤ 0.05) increase in MDA (nmol/ml) and PCC (nmol/ml) serum contents while reduced (*P* ≤ 0.05) GSH (nmol/ml), as compared to the control rams (Fig. 2). The addition of dietary *N. oculata* 4% to Cd and Pb intoxicated rams (G5 and G6) resulted in significant (*P* ≤ 0.05) amelioration of MDA, PCC, and GSH levels in comparison to Cd (G3)- and Pb (G4)-exposed groups. Concerning the effect of *N. oculata* 4% as compared to the negative control group, non-significant changes were recorded in PCC, on the other hand, significant (*P* ≤ 0.05) differences in MDA and GSH were observed (Fig. 2).

### Principal Component Analysis

**Fig. 3:**
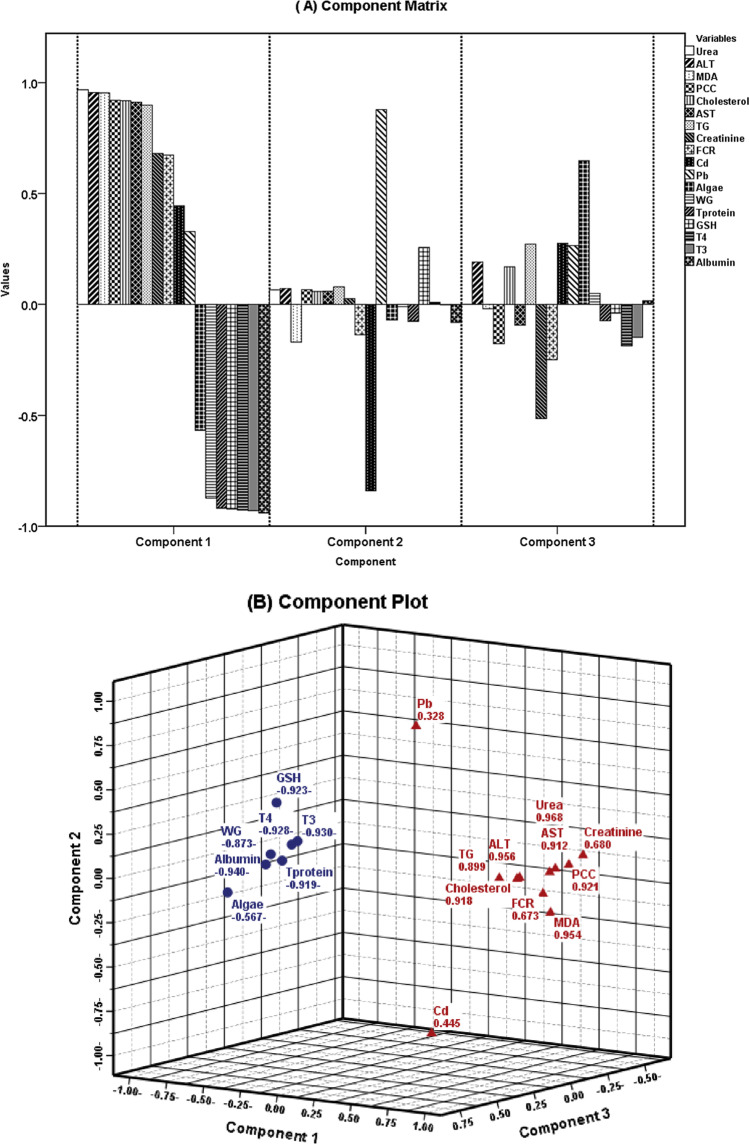
Principal component analysis of cadmium or lead intoxication and *N. oculata* 4% supplementation with growth performance, liver and kidney markers, biochemical parameters, and antioxidant markers in Barki rams. (A) Component matrix: the explained variance % was 70.588, 9.105, and 6.333%, while cumulative % was 70.588, 79.693, and 86.026% for components (PC1; PC2, and PC3, respectively). (B) Component plot: represent the principal component 1 variable interaction

To clarify the corrective effects of *N. oculata* 4% administration on Cd or Pb toxicity in an interactive manner, principal component analysis (linear correlation) was carried out on the former biomarkers (Fig. 3 A and B). Concerning the interactive effect with Cd or Pb toxicity, the parameters yielded three principal components (PC) that explained 87.2% of the total variances (Fig. 3A). PC1 had positive loading with both Cd and Pb, which correlated in a strong loading with Urea > ALT > MDA > PCC > cholesterol > AST > TG > creatinine > FCR were reported. Algae, on the other hand, supported the following parameters: total protein, albumin, GSH, T3, T4, and weight gain, which were directly correlated with them in the form of strong loadings (Fig. 3B).

## Discussion

Rams were chosen as the experimental animals in this study because they are an excellent model for ruminants and the ease of blood collection. Sheep, on the other hand, are more likely to expose to heavy metals since they graze herbage so close to the ground. As a result, it is hard to eliminate heavy metal exposure, and supplementation with *N. oculata* 4% instead may alleviate this impact in addition to its nutritional qualities. Furthermore, Altomonte et al. [41] hypothesized that ruminants would be appealing targets for this novel feedstuff since they can use non-protein nitrogen contained in algae and break down algal cell walls. Despite the potential benefits of using microalgae in ruminant nutrition, our current knowledge of the applications is limited. As a direct consequence, this study was conducted to investigate the reinforcing effect of *N. oculata* 4% as a novel natural feed supplement on rams’ performance, thyroid hormones, and some biochemical and antioxidative parameters, as well as its mitigating role against these parameters. The primary drawback is the high manufacturing cost [55], which makes them an uncompetitive feed choice [42]. The scenario may be altered shortly as a result of technological advancement [55].

Herein, rams were given an oral daily low metallic salt to repeat a feed exposure to detect Cd and Pb concentrations in the blood without causing clinical intoxication. The specified dose of 1 mg Cd and 5 mg Pb kg^−1^ body weight (33.6 kg) is comparable to the contaminated ruminant feed dose of 22.4 mg Cd and 112 mg Pb kg^−1^, the dose for a daily consumption of 1.5 kg of forage in sheep. It is worth noting that the absorption of Pb as acetate and carbonate is minimal and may reach 10% of intake due to the formation of insoluble complexes of lead in the gastrointestinal tract that are excreted with feces [56]. As a result, the predictable absorbable Cd and Pb dose lay within the tolerated concentrations in animal feed (0.5 Cd and 30 Pb mg kg^−1^), according to Liu [57]. Our analysis demonstrated that there were no external indicators of apparent toxicity in the exposed group. Clinical symptoms are not usually associated with blood element concentrations [56]. Furthermore, sheep may take up to 5 Pb mg kg^−1^ body weight for up to a year without showing any clinical signs [58]. Also, the lethal dose of Cd shows visible signs when the diet contains > 40 mg of Cd kg^−1^ of DM [58]. Cd toxicity is mostly determined by the organism’s mineral inflow, exposed dose, a chemical form of the metal, exposure time, species, and age [59].

According to the results of this study, Cd or Pb had a negative impact on sheep performance, which was confirmed by an inverse association between heavy metal administration and weight gain. This finding is consistent with the findings of Lane et al. [7] who found that some co-exposed cattle to Cd and Pb had the poor general condition. Concurrently, various publications have underlined Cd’s deleterious effects on growth rates in growing ruminants [60, 61]. Cd-exposed animals were recorded to exhibit a decrease in growth, weight gain, and food intake [10]. Pb intoxication is one of the most common types of toxicity in pastured animals [62], and its toxicity has been recorded in domestic animals; ruminants demonstrated greater settling and absorption of Pb in the reticulum; therefore, the absorbed Pb displaces some bivalent cations such as calcium and affects enzyme function [57].

The detected levels of Cd and Pb in the serum of the heavy metals’ corresponding exposed groups were only recorded after 60 days in low concentrations, which might be attributable to inadequate absorption of these elements from the gastrointestinal tract. Oral Cd and Pb absorption in the sheep is as low as 5% and 1.3%, respectively [63, 64]. The normal Pb level in bovine blood is 0.05 to 0.25 mg l^−1^ [65]. Intoxicated animals’ Pb levels in the blood can be restored to normal, but it requires time. This duration may range between 68 and 266 days, according to Miranda et al. [66]. This difference in returning to normal blood Pb levels (˂ 0.050 mg l^−1^) could be due to variation in lead absorbed and its particle size [66].

Regarding the significant decrease in serum T3 and T4 concentrations due to exposure to both elements, thyroid dysfunction may be related to structural damage of thyroid follicular cells caused by Cd and Pb accumulation in the thyroid gland, resulting in subclinical hypothyroidism [45, 46]. Yoshizuka et al. [67] hypothesized that Cd accumulating in the mitochondria of thyroid follicular epithelial cells may disrupt oxidative phosphorylation of this organelle and that the loss of energy supply may have inhibited thyroid hormone synthesis and release. Similarly, Pb can imply a decrease in T4 production and/or secretion from thyroid follicular cells [45, 46]. T3 is the active form of thyroid hormone; nevertheless, it accounts for just 20% of the hormone released; most of T3 is synthesized by the peripheral conversion of T4 to T3, whereas T4 accounts for more than 80% of the hormone secreted [68]. The peripheral deiodination of T4 to T3, which occurs primarily in the liver, is dependent on the activity of 5′-monodeiodinase (5′-D) [69, 70]. Studies have also found that Cd and Pb interfere with thyroid function at both the glandular and peripheral levels by preventing the conversion of T4 to T3 [45, 46]. A significant decrease in thyroid hormones was reported after the dosage of Pb [71] in buffalo cows, meanwhile, Zongping et al. [72] observed an increase in thyroid hormones of the sheep had high blood Pb concentrations. Such change in thyroid hormones may be related to high dosage and long duration of exposure to Pb [24]. Similarly, hepatic pathology affects serum thyroid hormone concentrations as a result of the impacts on peripheral enzyme pathways [73]. As a result, a partial drop in serum T3 concentrations in Cd and Pb-treated sheep may be associated with hepatic dysfunction [45, 46].

The most plausible explanation for the increase in hepatic enzymatic activities and renal markers is that Cd and Pb have detrimental impacts on liver and kidney tissues, releasing intracellular enzymes into the bloodstream [2], as well as increased cellular metabolic rate, irritability, and liver damage [74]. High AST and ALT activities are associated with increased liver microsomal membrane fluidity, free radical production, and liver tissue alteration [75]. This is supported by the significantly positive loading of Cd and Pb with serum former parameters as shown in PCA. Badiei et al. [45] reported increased levels of ALT and AST in experimentally Pb-poisoned Iranian rams [76] and confirmed comparable results in the Merino sheep. Similar outcomes were observed after oral administration of Pb in goats [77] and sheep [78].

The liver is considered one of the body’s key metabolic organs, regulating and maintaining lipid homeostasis [79]. As a result, increasing blood lipid levels could be attributed to increased lipoprotein production or reduced lipoprotein clearance [80]. The current study revealed a significant increase in cholesterol (mg dl^−1^) and TG (mg dl^−1^) levels in serum samples of Cd-intoxicated sheep, which is consistent with the findings of Chowdhury et al. [81] who found a significant increase in rats treated with Cd chloride, as well as alterations in the lipid profile and total cholesterol in Cd-administered animals. As a sequence, the high serum lipid levels could be attributed to increased synthesis or impaired clearance of lipoproteins. This result could be explained by the impairment of liver function induced by an imbalance in the antioxidant defense system in Cd-intoxicated rats; the Cd toxic state lowered HDL synthesis in the liver [82]. Furthermore, Cd toxicity causes a variety of derangements in lipid metabolic and regulatory processes, which leads to dyslipidemia, the most common metabolic complication observed in heavy metal toxicity, which is characterized by distinct changes from a normal plasma lipid and lipoprotein profile [83]. A similar profile as in the Cd-exposed group was observed in the Pb-exposed group; however, decreased clearance of lipoproteins may occur as a result of changes in the cell-surface receptors for lipoprotein [84] or as a result of suppression of hepatic lipoprotein lipase activity [85]. Furthermore, Pb has been demonstrated to inhibit the activity of cytochrome P450 [86], which can restrict the production of bile acids, which is the major pathway for cholesterol removal from the body.

In the present study, the significant decrease in serum proteins of Cd- or Pb-exposed groups compared could be induced by several pathological processes caused by heavy metals, including plasma dissolution, renal damage, and protein elimination in the urine, a decrease in liver protein synthesis due to hepatic damage, and changes in hepatic blood flow and/or hemorrhage into the peritoneal cavity and intestine [24]. Prabu et al. [87] found that Cd-exposed rats had reduced plasma total protein, albumin, and globulin levels. Similarly, when lambs were exposed to various levels of Pb, similar outcomes were obtained [88].

Regarding serum oxidative stress markers in the present study, there was a positive significant correlation between Cd and Pb exposure and MDA level. A significant increase in serum MDA levels of Pb-exposed group is in accordance with those previously mentioned by Kanter et al. [89] and Bayoumi et al. [90]. Such elevation could be attributed to Pb-induced lipid peroxidation, and the strong positive correlations between serum MDA and Pb administration [91]. A significant reduction in GSH was observed in the Cd- and Pb-exposed groups, as well as a significant negative correlation between them. These findings are consistent with those documented by Oraby et al. [11]. This result augmented the existence of oxidative stress whereas, the oxidative-stress-caused damage of macromolecules other than lipids; as a result, ROS can damage multiple biological molecules [92], and indices of lipid peroxidation may never be adequate markers of cellular damage caused by oxidative stress [93]. For this reason, the protein carbonyl content (PCC) in blood was evaluated as a marker of oxidative protein damage [92], as it is universally recognized as a gold standard for identifying protein oxidation [94]. It is an irreversible oxidative protein modification that is assumed to be an early indicator of protein oxidative stress-related disorders [95]. Metal-catalyzed oxidation of lysin, proline, arginine, and threonine residues, direct oxidation of tryptophan, and reactive lipid peroxidation products of cysteine, histidine, and lysine can all result in the formation of protein carbonyls [95]**.** The current study’s findings of a negative correlation between serum albumin and PCC levels in the Cd- and Pb-exposed groups could support the well-established theory that a low serum albumin level indicates the presence of systemic inflammation and oxidative stress [96].

Herein, a 4% *N. oculata*-supplemented group compared to a control group showed no effect on serum T3, ALT, AST, cholesterol, and TG. Furthermore, these findings indicate normal activity and low impacts on liver function, showing the superior safety of feeding *N. oculata* to rams. The results also show the unaffected release of triglyceride-rich lipoproteins into the lymphatic system, which is consistent with the results of unaffected daily bodyweight reduction. Furthermore, the prior results are consistent with those published by Kholif et al. [42]. Rams in the microalga-supplemented group had the lowest significant MDA content and the highest GSH level among the treated groups. In general, *Nannochloropsis* is a rich source of proteins and lipids with an excellent fatty acid profile; the consumption of eicosapentaenoic acid (EPA) and other polyunsaturated fatty acids (PUFAs) is the most essential element of value for this microalga, which is supplemented by a significant contribution of other anti-oxidant components with high biological activity, including polyphenols, carotenoids, and vitamins [34].

The role of *N. oculata* 4% supplementation to mitigate the Cd or Pb exposure was pronounced, with decreasing serum levels of both elements and significant improvements in weight gain and FCR reported in these groups when compared to heavy metal-exposed groups. A possible explanation is that microalga has accelerated extracellular passive adsorption (biosorption) and slow intracellular positive dispersion and buildup (bioaccumulation) with heavy metals, in furthermore to cell polymeric substances like peptides and exopolysaccharides with uronic groups; the cell wall of microalga is primarily composed of polysaccharides (cellulose and alginate), lipids, and organic proteins, which provide many functional groups (such as amino, hydroxyl, carboxyl, phosphate, imidazole, sulfonate, thiol, and others) capable of binding heavy metals [97].

Considering that *Nannochloropsis* is a high source of omega-3 fatty acids, particularly eicosapentaenoic acid (EPA) [40], there may be a link between *Nannochloropsis* supplementation and thyroid hormone levels, as hypothesized by Makino et al. [98], who claims that administration of EPA-E prevents a decrease in thyroid hormone levels, as omega-3 (polyunsaturated fatty acid (PUFA), containing EPA) controls thyroid cell activity via two major processes: signal transduction channel modification by modifying membrane fatty acid composition; and fast, direct stimulation of gene transcription. Additionally, *N. oculata* 4% supplementation, significantly increased both T3 and T4 in Cd- or Pb-exposed groups with microalga supplementation, as compared to the heavy metal exposed groups. The current study performed PCA to analyze the detailed interaction between thyroid hormones and liver enzymes, which demonstrated a highly significant inverse correlation; also, microalga exhibited a direct relationship with thyroid hormones and an inverse association with liver enzymes. Moreover, similarly, hepatic pathology affects serum thyroid hormone concentrations as a result of the impacts on peripheral enzyme pathways [73]. As a result, a partial drop in serum T3 concentrations in Cd- and Pb-treated sheep may be associated with hepatic dysfunction [45, 46]. Also, the correction of Cd or Pb exposure by *N. oculata* 4% supplementation was supported by the positive correlation of microalga supplementation with serum protein parameters and the negative correlation with liver enzymes and kidney function markers; this finding is partially in agreement with Aboulthana et al. [99] and Nacer et al. [100] who stated that a diet supplemented with microalga *N. gaditana* and *N. oculata* provided good protection against renal dysfunction in diabetic rats because this alga has great potential to normalize the contents of serum uric acid, urea, and creatinine in rats with diabetes. Also, Nacer et al. [100] added that *N. gaditana* caused a reduction in the activity of AST and ALT enzymes, which indicated their hepatoprotective effect.

Likewise*, N. oculata’s* hypocholesterolemic action in heavy metal-exposed groups may be attributed to the inhibition of cholesterol absorption from the intestines [101] or suppression of oxidation and LDL-C uptake [102]. Also, *N. oculata* can change bile acid absorption and metabolism, or increase propionic acid generation as a result of the fermentation of the soluble fiber content in the algal residue with an increase in this short-chain fatty acid (SCFA), which also hindered hepatic cholesterol synthesis [103]. Furthermore, Markovits et al. [104] revealed that dietary fibers found in *Nannochloropsis*, particularly insoluble fibers, inhibit intestinal cholesterol absorption and have an anti-hypercholesterolemic effect [105]. It was also proven that *N. gaditana* can improve lipid metabolism [106].

MDA and PCC showed a significant decrease in Cd or Pb with microalga supplementation groups whereas GSH contents were significantly higher than those in heavy metal-intoxicated groups. Simultaneously, PCA analysis revealed that microalga supplementation was negatively correlated with MDA and highly correlated with the previously mentioned antioxidant markers, suggesting that the microalga ameliorate the disruption of anti-oxidative defense mechanisms, implying a potential therapeutic role. *N. oculata* contains a high content of -3 PUFAs (-linolenic, ALA, C18:3 3) [107] and eicosapentaenoic (EPA, C20:5 3) [108], indicating that PUFAs may significantly contribute to its antioxidant capacity. Furthermore, carotenoids from *N. oculata* have similar antioxidant activity [109].

## Conclusion

In conclusion, *N. oculata* as a feed supplement (4%) improves renal activity (creatinine and urea), T4, and oxidative stress indicators as compared to the control group. The impact of Cd or Pb administration was observed in all the examined parameters, which were significantly different from the control. Animals exposed to Cd or Pb and supplemented with microalga significantly outperformed in the measured parameters than heavy metal-exposed groups. The administration of *N. oculata* (4%) might help to mitigate the oxidative stress and endocrine disruptive induced by Pb and Cd exposure.

## Data Availability

All the data generated or analyzed during this study are included in this published article (and its supplementary information files).
