# Correction to: Evaluation of Cadmium or Lead Exposure with Nannochloropsis oculata Mitigation on Productive Performance, Biochemical, and Oxidative Stress Biomarkers in Barki Rams

**DOI:** 10.1007/s12011-022-03342-z

**Published:** 2022-07-01

**Authors:** Marwa A. Hassan, Yasmina K. Mahmoud, A. A. S. Elnabtiti, A. S. El-Hawy, Moharram Fouad El-Bassiony, Heba M. A. Abdelrazek

**Affiliations:** 1 Department of Animal Hygiene, Zoonoses and Behavior, Faculty of Veterinary Medicine, Suez Canal University, Ismailia, 41522 Egypt; 2 Biochemistry Department, Faculty of Veterinary Medicine, Suez Canal University, Ismailia, 41522 Egypt; 3 Animal Wealth Development Department, Faculty of Veterinary Medicine, Suez Canal University, Ismailia, 41522 Egypt; 4 Animal and Poultry Production Division, Desert Research Center, Cairo, Egypt; 5 Department of Physiology, Faculty of Veterinary Medicine, Suez Canal University, Ismailia, 41522 Egypt

**Correction to: Biological Trace Element Research**

https://doi.org/10.1007/s12011-022-03318-z

The original version of this article unfortunately contained a mistake. The name of “Moharram Fouad El-Bassiony” is now spelled out in the author group.

The original article has been corrected.

